# Gut Microbiome Reveals Specific Dysbiosis in Primary Osteoporosis

**DOI:** 10.3389/fcimb.2020.00160

**Published:** 2020-04-21

**Authors:** Zhiming Xu, Zheng Xie, Jianguang Sun, Shilei Huang, Yangyang Chen, Chengxiang Li, Xing Sun, Bicheng Xia, Lingyang Tian, Caijuan Guo, Feng Li, Guofu Pi

**Affiliations:** Department of Orthopaedics, First Affiliated Hospital of Zhengzhou University, Zhengzhou University, Zhengzhou, China

**Keywords:** primary osteoporosis, gut microbiome, 16s rDNA, metagenomic analysis, biomarker

## Abstract

**Object:** Primary osteoporosis (PO) is the most common bone disease, which is characterized by decreased bone mass, damage of bone tissue microstructure, increased bone fragility, and is prone to fracture. Gut microbiome may be involved in bone metabolism of PO through gut–brain axis regulation of immune system and endocrine system, however, the specific mechanism is still unclear. The purpose of this study was to characterize the gut microbiome of patients with PO and its possible role in the occurrence and development of the disease.

**Methods:** Fecal samples were collected from 48 PO patients and 48 healthy controls (HC). The composition of gut microbiome community was analyzed by 16s rDNA amplification sequencing, and the difference of gut microbiome composition between PO patients and HC individuals was compared. PICRUSt was also used to predict the biological function of gut microbiome in patients with PO, and to explore its possible role in the occurrence and development of this disease. The classification model is constructed by random forest algorithm so as to screen the key biomarkers.

**Result:** The diversity of gut microorganisms in PO patients was significantly higher than that in HC group (*p* < 0.05) and there was significant difference in microbial composition in PO group. The abundance of Dialister (0.036 vs. 0.004, *p* < 0.001) and Faecalibacterium (0.331 vs. 0.132, *p* < 0.001) were significantly enriched which were the key flora related to PO. Although no significant correlation between bone mineral density and the richness of microbial communities are found, PICRUST results show that there are a wide range of potential pathways between gut microbiome and PO patients, including genetic information processing, metabolism, environmental information processing, cellular processes, human diseases, and organic systems. Notably, the discriminant model based on dominant microflora can effectively distinguish PO from HC (AUC = 93.56).

**Conclusions:** The findings show that PO is related to the change of gut microbiome, especially the enriched Dialister and Faecalibacterium genera, which give new clues to understand the disease and provide markers for the diagnosis and new strategies for intervention treatment of the disease.

## Introduction

Osteoporosis, the most common bone disease, which is characterized by decreased bone mass, damage of bone tissue microstructure, increased bone fragility and prone to fracture (Ensrud and Crandall, 2017), can be divided into two categories: PO and secondary osteoporosis. Additionally, PO includes postmenopausal osteoporosis, senile osteoporosis, and idiopathic osteoporosis (Brown, 2017), a skeletal disease associated with aging. With the aging of the social population, the incidence of osteoporosis and osteoporotic fracture is increasing, which has become an important public health problem in China (Wang et al., 2009). Osteoporotic fracture refers to a fracture that occurs during minor trauma or daily activities, which is a serious consequence of osteoporosis (Qaseem et al., 2017), one of the main causes of disability and death among elderly patients (Black and Rosen, 2016). Moreover, the medical treatment and care of osteoporosis and fractures require a lot of human, material and financial resources, resulting in a heavy family and social burden (Cairoli et al., 2015).

The integrity of bone is maintained by repeated, spatio-temporal coupling of bone resorption and bone formation, which leads to bone loss and osteoporosis when there is a negative balance between bone formation and bone resorption (Ensrud and Crandall, 2017). While, the regulation of bone metabolism is affected by endocrine factors and a variety of cytokines (Henriksen et al., 2015). Studies have shown that abnormal gut microbiome is associated with more and more health status (Sekirov et al., 2010; Kundu et al., 2017; Song et al., 2018). Related basic and clinical studies have shown that gut microbiome may be involved in bone metabolism through gut-brain axis regulation of immune system, endocrine system, and so on (Ohlsson and Sjögren, 2015; Yan and Charles, 2017; Ibáñez et al., 2019). A large number of studies have shown that there is an interaction between solid hormones and gut microbiome (Baker et al., 2017). In animal experiments and clinical trials, it has been confirmed that gut microbiome can affect the synthesis and decomposition of adrenal glucocorticoid (Shahi et al., 2017; Lee et al., 2019), finding that gut microbiome can affect bone metabolism by regulating the immune state of the body (Hernandez et al., 2016), in addition, gut microbiome can also affect bone metabolism through Wnt/β-catenin, Th17, Tregs, and NOD signaling pathways (Lee et al., 2019). Recent animal studies in aseptic mice have shown that gut microbiome is also a regulator of bone mass (Ohlsson and Sjögren, 2015). Although the previous small sample size study showed differences in the composition and quantity of gut microbiota between PO patients and HC patients (Wang et al., 2017), sufficient sample size studies have not been conducted to assess the association between osteoporosis and microbiota. The mechanism by which intestinal flora regulates bone metabolism needs further study (Yan and Charles, 2017).

In this study, we carried out the 16S rDNA amplicon sequencing of intestinal flora in patients with PO (48 cases) and a group of age-and sex-matched HC (48 cases). Based on the in-depth study of the microbial components related to the disease, the differences between the composition of gut microbiome in patients with PO and HC group were obtained, and these data were correlated with clinical parameters. In order to understand the community structure deeply, the function of microbial community in patients with PO was predicted. This study provides a theoretical basis for the relationship between gut microbiome and the incidence of PO, and provides a new strategy for the treatment of patients with PO.

## Materials and Methods

### Study Subjects

The prospective study was approved by the Institutional Review Board of the First Affiliated Hospital of Zhengzhou University, with all participants signing informed consent forms. A total of 96 stool samples from inpatients in our hospital in 2019 were prospectively collected in this study. All participants were orthopedic inpatients, and the patients were selected according to the exclusion criteria: (1) secondary osteoporosis; (2) incomplete case data or patients and their families could not cooperate to complete the questionnaire survey; (3) age <40 years old; (4) antibiotics, probiotics, probiotics, or symbiosis in the 2 months before stool samples were collected; (5) patients with severe malnutrition, infection, drug use, or alcohol abuse; (6) patients with digestive system diseases; (7) patients with severe systemic diseases, tumors, or other serious primary diseases; (8) previous lumbar and hip surgery; (9) fecal samples were not up to standard (Lorentzon and Cummings, 2015). The clinical data, demographic data and eating habits of the participants were collected through hospital electronic medical records and questionnaires. After the specimens were collected, they were screened again, 48 cases of PO and 48 cases of HC were finally included in the study and received 16s rDNA Amplicon Sequencing.

### Clinical Data Collection

The age, sex, smoking history, drinking history, and eating habits of the participants were collected by questionnaire. The researchers measured the height and weight of each participant and calculated the body mass index (BMI). The bone mineral density of spine (BMDs) and bone mineral density of hip (BMDh) were measured by dual energy X-ray absorptiometry (Discovery DXA, HOLOGIC, USA) (Lorentzon and Cummings, 2015). The diagnosis of primary osteoporosis was based on the diagnostic criteria recommended by WHO and based on the results of DXA measurement: the bone mineral density was lower than that of the same gender, and the bone peak value of healthy adults of the same race was normal. The reduction of 1 × 2.5 standard deviation was low bone mass (or low bone mass), and the decrease of 2.5 standard deviation was osteoporosis. The degree of bone mineral density decreased in accordance with the diagnostic criteria of osteoporosis, accompanied by one or more fragile fractures for severe osteoporosis.

### Fecal Sample Collection, DNA Extraction and PCR Amplification

Each participant was required to use a sterile collection container to collect fresh fecal samples at 06:30–08:30, then placed in an icebox and transferred to the laboratory within 2 h, which is packaged into 2 2 ml cryopreservation tubes and frozen at −80°C until DNA extraction. The genomic DNA of the sample was extracted by CTAB method, and then the purity and concentration of the extracted DNA were detected by agarose gel electrophoresis. The sample was diluted to 1 ng/ μ l with sterile water, then, using diluted genomic DNA as template, 515F and 806R primers with Barcode, Phusion High-Fidelity PCR Master Mix with GC Buffer (New England Biolabs, USA) and high efficiency high fidelity enzyme were used to amplify the 16S rDNA V3-V4 region of bacteria. Two percentage of agarose gel was used to detect PCR products. After the PCR products were fully mixed, the PCR products were purified by agarose gel electrophoresis with 1 × TAE concentration of 2%. The target bands were cut and the products were recovered by GeneJET gel (Thermo Scientific, USA) recovery kit. Then the library was constructed by Ion Plus Fragment Library Kit 48 rxns library construction kit (Thermofisher, USA), and the constructed library was detected by Qubit quantitative and library detection. After the library is qualified, Ion S5TMXL (Thermofisher, USA) is used for computer sequencing according to the recommendation of the reagent.

### OTU Clustering and Species Annotation

First, the low quality part of reads was cut by Cutadapt (V1.9.1), then the sample data were separated from the obtained reads according to Barcode, and the Barcode and primer sequences were truncated to obtain Raw reads. Finally, the Reads sequence was compared with the species annotation database, the chimera sequence was detected and removed, and the Clean Reads were obtained.

All the Clean Reads of all samples were clustered by Uparse software (Uparse v7.0.1001). The sequences were clustered into Operational Taxonomic Units (OTUs), with 97% identity and the sequences with the highest frequency were taken as the representative sequences of OTUs according to the principle of algorithm. Then the species annotation of OTUs sequence was analyzed by Mothur method and SILVA132 SSUrRNA database (Set the threshold to 0.8–1), and the taxonomic information was obtained. At the same time, the bacterial community composition of each sample at each classification level (kingdom, phylum, class, order, family, genus, species) was calculated. After that, the phylogenetic relationship of all OTUs sequences is obtained by using MUSCLE (Version 3.8.31) software for fast multi-sequence alignment. Finally, the sample with the least amount of data is taken as the standard to homogenize the data of each sample. The homogenized data were followed by sample complexity analysis (Alpha diversity analysis) and sample comparative analysis (Beta diversity analysis).

### Analysis on Gut Microbiome

Alpha Diversity is used to analyze the diversity of microbial community in Within-community. The diversity analysis of single sample can reflect the richness and diversity of microbial community in the sample. The composition of microbial community in two groups of different samples was compared and analyzed by Beta Diversity. First of all, according to the species annotation results of all samples and the abundance information of OTUs, the OTUs information of the same classification is combined to obtain the species abundance information Profiling Table. At the same time, using the phylogenetic relationship between OTUs, the Unweighted Unifrac, is further calculated to obtain a distance matrix. Then, the Weighted Unifrac distance is further constructed by using the abundance information of OTUs to Unweighted Unifrac. Finally, the differences of microflora between PO group and HC group were analyzed by means of Principal Component Analysis (PCA), Principal Co-ordinates Analysis (PCoA), Non-Metric Multi-Dimensional Scaling (NMDS), Unweighted Pair-group Method with Arithmetic Means (UPGMA) and Beta diversity index.

Spearman correlation analysis was used to analyze the relationship between clinical data and microbial richness, so as to obtain the correlation and significant *P*-value between each data and microbial abundance. Random forest algorithm was used to construct the classification model and screen the Biomarker that plays an important role in grouping. Based on the analysis of random forests with species abundance, different numbers of species were selected according to different classification levels to construct a random forest model. The important Biomarkers were screened by MeanDecreaseAccuracy and MeanDecreaseGin, and then cross validation conducted for each model (default 10-fold).

### Analysis on Microflora Structure Differences Between Groups

Anosim analysis was used to test whether the difference between groups was significantly larger than that within groups, so as to judge whether the grouping was meaningful or not, which is based on the rank of Bray-Curtis distance value for inter-group difference significance test, using R vegan package anosim function. *T*-test was carried out between the two groups to find out the species with significant differences between the two groups at each classification level (*p* < 0.05). The MetaStat method was used to test the species abundance data between groups to get the *p*-value, and the *q*-value was obtained by correcting the *p*-value. Finally, the species with significant differences were selected according to the *q*-value. LEfSe (LDA Effect Size) was used to compare the two groups to find out the Biomarker with statistical difference between the two groups, which is an analytical tool for discovering and interpreting high-dimensional biometric markers (genes, pathways, and taxa), emphasizing statistical significance and biological relevance, and is able to identify the characteristics of different abundance and associated categories.

### Metagenomic Function Prediction

PICRUSt can perform metagenome function prediction based on KEGG database according to 16S rDNA sequencing data, which, based on the OTU tree in the Greengene database, and the genetic information on the OTU, infers the gene function spectrum of their common ancestors, and the gene function spectrum of other untested species in the Greengenes database so as to construct the archaeal and bacterial domain full spectrum. The gene function predicts the spectrum, and finally the sequence composition obtained by sequencing is matched with the database to predict the metabolic function of the flora.

## Result

### Clinical Data Characteristics

In this study, 139 stool samples were prospectively collected from inpatients in the First Affiliated Hospital of Zhengzhou University. After strict clinical diagnosis and exclusion, 96 cases were included in this study, which were divided into primary osteoporosis group (48 cases in total, female 29 cases) and HC group (48 cases in total, female 30 cases). The patients of the two groups were matched with regard to the age, gender, body mass index, and eating habits. There was a significant difference in BMD of lumbar vertebrae and hips between patients with osteoporosis and healthy controls (*p* < 0.01) (Table 1).

**Table 1:** Clinical characteristics of the participants.

**Clinical indexes**	**HC (*n* = 48)**	**PO (*n* = 48)**	***P*-values**
Age (year, mean ± SD)	60.23 ± 11.43	57.50 ± 10.15	0.219
Gender			
Female (%)	30 (62.5%)	29 (60.42%)	0.834
Male (%)	18 (37.5%)	19 (39.58%)	
BMI (kg/m^2^, mean ± SD)	25.83 ± 3.83	25.72 ± 3.61	0.883
BMDs (g/m^3^, mean ± SD)	1.09 ± 0.13	0.79 ± 0.10	<0.01
BMDh (g/m^3^, mean ± SD)	0.98 ± 0.10	0.73 ± 0.12	<0.01
Dietary habit	Mixed diet	Mixed diet	–

*The independent sample T-test was used to compare the continuous variables; Chi-square exact test compared categorical variables. HC, healthy control; PO, primary osteoporosis; BMI, body mass index; BMDs, lumbar bone mineral density; BMDh, hip bone mineral density*.

### Increased Microbiome Abundance in PO Patients

**Figure 1:**
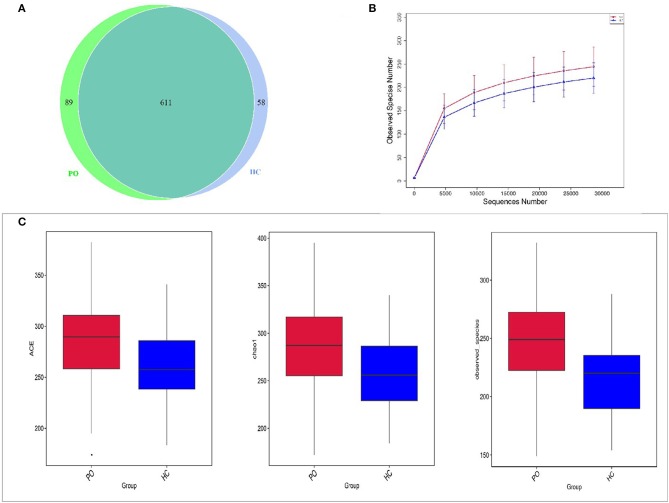
Comparison of gut microbiome structure between PO and HC group. **(A)** Venn diagram: each circle in the figure represents a group, the number of overlapping parts represents the number of OTUs shared between the groups, and the number without overlapping parts represents the number of OTUs unique to the group. **(B)** Rarefaction Curve, Abscissa is the number of sequencing strips randomly selected from a sample, and ordinate is the number of OTU that can be constructed based on the number of sequencing strips, which is used to reflect the sequencing depth (Blue, HC; Red, PO). **(C)** The alpha diversity of the two groups of gut microbial communities was described according to the ACE, Chao1 and observed_species indices. Box plot reflects median, degree of dispersion, maximum, minimum, and outliers. *P*-values were determined using *T*-test and wilcox Rank Sum Test. HC, healthy control; PO, primary osteoporosis; OTU, operational taxonomic unit.

**Figure 2:**
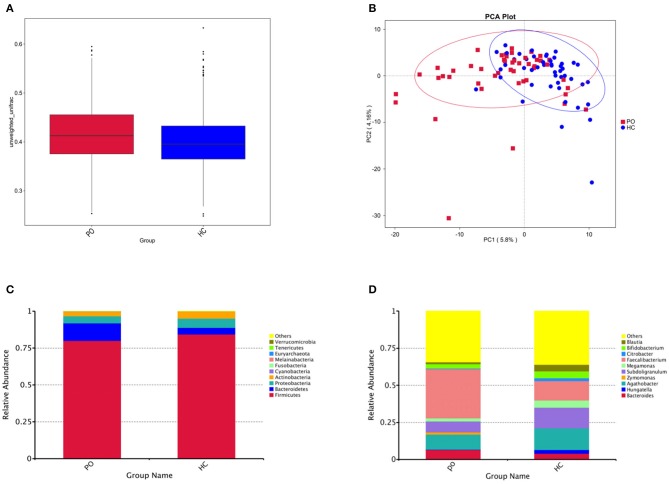
**(A)** Box chart based on Beta diversity showed significant difference between the two groups. Abscissa represents Weighted Unifrac, Box plot response median, degree of dispersion, maximum, minimum, outliers. **(B)** Principal Component Analysis (PCA), the abscissa represents the first principal component, the percentage represents the contribution of the first principal component to the sample difference; the ordinate represents the second principal component, and the percentage represents the contribution of the second principal component to the sample difference; each point in the graph represents A sample (PC1 = 5.8%; PC2 = 4.16%) (Blue, HC; Red, PO). Cylindrical accumulation Map of relative abundance of species at Phylum level **(C)** and Genus level **(D)**. The abscissa is grouping information; the ordinate represents Relative Abundance; others represents the sum of the relative abundances of all the phylums except the 10 phylums in the figure.

In order to study the composition and function of gut microbiome in PO patients, high throughput sequencing of 16s rDNA gene V3-V4 region was performed in 96 fecal samples. The Effective Tags of all samples were clustered by OTUs with 97% identity, and a total of 758 OTUs were obtained, of which, there were 611 of the two groups, 89 OTUs were unique to the PO group, and 58 were unique to the HC group (Figure 1A). The results of Rarefaction Curve analysis showed that the dilution curve of PO group and HC group tended to be flat, indicating that the amount of sequencing data was gradually reasonable, and more data would only produce a small number of new species (OTUs). Therefore, the sample size of the study is sufficient, the sequencing depth is up to the standard, and there is no need for additional sample size (Figure 1B). Although there was no significant difference in shannon index and simpson index between the two groups, chao1 (PO vs. HC: 286.29 vs. 259.09, *P* = 0.004) and ACE (PO vs. HC: 284.27 vs. 263.47, *P* = 0.012) and observed_species (PO vs. HC: 220 vs. 244, *P* = 0.002) suggested that the intestinal microbial diversity in primary osteoporosis was significantly higher than that in HC group (Figure 1C). The diversity of Beta was analyzed based on Weighted Unifrac and Unweighted Unifrac. Anosim analysis (R = 0.15, *P* < 0.001), permutational MANOVA (R = 0.06, *P* < 0.001) suggested that there were significant differences between the two groups. Importantly, there was also a illustrious discrepancy in the composition of gut microbiome between the PO group and the HC group (MRPP, A = 0.0314, observed-delta = 0.7193, expected-delta = 0.7426, *P* = 0.001) in the Bray-Curtis-based PCA diagram (Figures 2A,B).

### Alterations of Microbiomes in PO Patients

**Figure 3:**
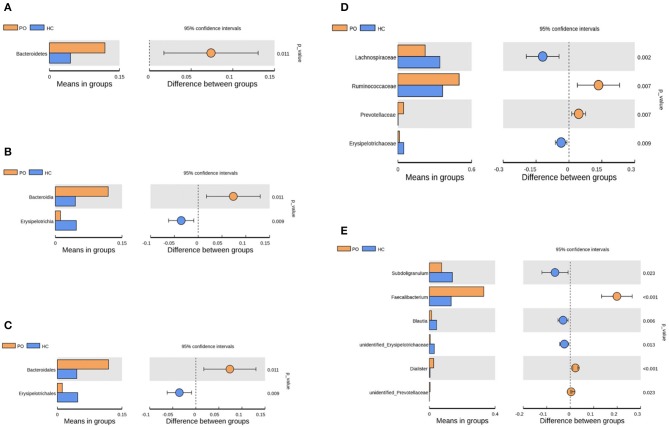
Species analysis of differences between groups by *T*-test between groups. Differential species between the two groups at the phylum **(A)**, class **(B)**, order **(C)**, family **(D)**, and Genus **(E)** classification levels. In each of the figures, the left panel shows the abundance of species differences between groups, and each bar in the graph represents the mean of each species in each group with significant differences in abundance between the groups. The graph on the right shows the difference between the confidence levels of the groups. The leftmost endpoint of each circle in the figure represents the 95% confidence interval lower limit of the mean difference, and the rightmost endpoint of the circle represents the 95% confidence interval upper limit of the mean difference. The center of the circle represents the difference in the mean. The group represented by the circle color is a group with a high mean. At the far right of the displayed results is the inter-group significance test *p*-value for the corresponding species (Orange, PO; Blue, HC).

**Figure 4:**
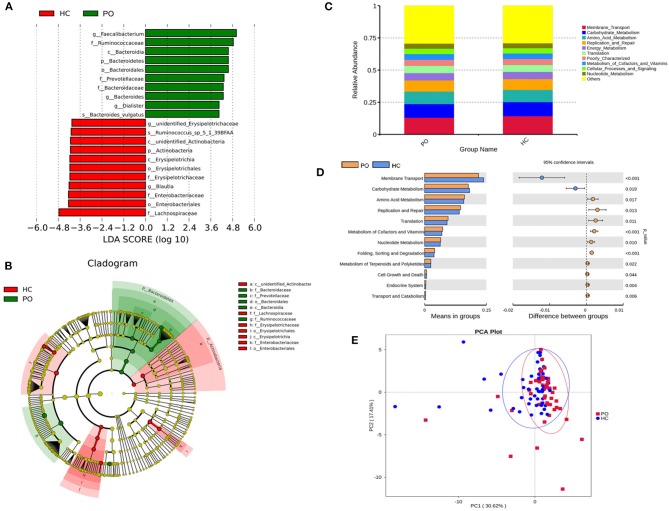
**(A)** The LDA value distribution histogram shows the species with an LDA score greater than the set value (the default setting is 4), that is, the Biomarker with statistical differences between groups. The length of the histogram represents the impact of the different species (LDA score). **(B)** Evolutionary bifurcation graph, the circle of radiation from the inside to the outside represents the classification level from the gate to the genus (or species). Each small circle at different classification levels represents a classification at that level, and the diameter of the small circle is proportional to the relative abundance. The coloring principle: the species with no significant difference were uniformly colored yellow, the differential species Biomarker followed the group, and the red nodes represent the microbial group which played an important role in the red group. Green nodes represent microbial groups that play an important role in the green group. Where one of the groups is missing, it shows that there are no significant differences in species in this group, so the group is missing. The name of the species represented by the English letters in the picture is shown in the illustration on the right. **(C)** Level 2 relative abundance column chart, abscissa is grouping information; ordinates represent the relative abundance of functions; others represents the sum of the relative abundance of all functions other than these 10 functions in the diagram. **(D)** Inter-group functional difference analysis graph (for the illustration, please refer to the inter-group species difference analysis chart). **(E)** Display of PCA results of PICRUSt functional comments (PC1 = 30.62%; PC2 = 17.41%).

Species annotation was accomplished on the representative sequence of OTUs, and the statistical analysis of community structure differences was carried out according to the results of species annotation, we found evident changes in gut microbiome structure in patients with PO at a wide range of phylogenetic levels. Applying the Simper analysis to the decomposition of the Bray-Curtis difference index and quantifying the contribution of each species to the difference between the two groups, it is found that the key microflora types were mainly distributed in the Firmicutes, Bacteroidetes, and Proteobacteria phylum. The species with the maximum abundance at the top 10 in each classification level were selected to produce the relative abundance column accumulation map of species, and the species relative abundance column accumulation map at phyla and genus level were shown in Figures 2C,D. *T*-test was implement between PO patients and HC groups. Compared with the control group, we found that the abundance of Bacteroidetes phylum, Bacterioidia class, and Bacteroidetes order in PO patients were significantly enriched at the corresponding phylum, class and order level (0.118 vs. 0.045, *p* = 0.011); at the level of class, order and family, the abundance of Erysipelotrichia group was significantly lower than that of HC group (0.012 vs. 0.047, *p* = 0.008). In addition, at the level of family, Ruminococcaceae and Prevotellaceae were significantly enriched in PO group, while the abundance of Lachnospiraceae in HC group was significantly lower than that in HC group. The abundance of Faecalibacterium (0.331 vs. 0.132, *p* < 0.001), unidentified_Prevotellaceae (0.004 vs. 0.001, *p* = 0.023) and Dialister (0.036 vs. 0.004, *p* < 0.001) increased significantly at genus level in patients with PO. The microflora abundance of Subdoligranulum (0.075 vs. 0.140, *p* = 0.023), Blautia (0.013 vs. 0.044, *p* = 0.006), and unidentified_Erysipelotrichaceae (0.005 vs. 0.030, *p* = 0.013) decreased, and the difference was statistically significant (Figure 3). Furthermore, we identified statistically significant microbes between the two groups by LEfSe analysis. It is noteworthy that the enriched Bacteroidetes phylum, Bacterioidia class, Bacteroidetes order, Ruminococcaceae family, Prevotellaceae family, Dialister genus, and Faecalibacterium genus have been revealed as the key microbes related to PO (Figures 4A,B).

### Predicted Function Analysis of Microbiome

In order to study whether the difference of intestinal microbial composition in patients with PO also had a functional effect on the expression of some genes, the researchers used PICRUST to predict the function of 16s sequencing data based on KEGG database, by which, a wide range of potential pathways were identified between intestinal flora and PO patients, including metabolism, genetic information processing, environmental information processing, cellular processes, organismal systems, and human diseases (Figure 4C). In Level 2 KEGG signaling pathway, the microbial function of PO patients involved in membrane transport and carbohydrate metabolism decreased, and the difference was statistically significant (*p* < 0.05). Compared with HC group, the homologous genes of PO patients increased significantly, including amino acid metabolism; cofactor and vitamin metabolism; nucleotide metabolism; terpenoid and polyketone metabolism; replication and repair, translation, folding, classification, and degradation in genetic information processing; transport and catabolism in cell processes; cell growth and death; biological system; endocrine system; immune system; digestive system; and immune system disease microbial function (Figure 4D). The results of PCA analysis also showed that the intestinal flora function of PO patients was different from that of HC group (Figure 4E). Obviously, 94 homologs in PO patients using Level 3 KEGG pathway were plainly different from those in the HC group. For example, transporters, ABC transporters and transcription factors homologs in PO patients significantly reduced, while the homologous genes of general function prediction only, ribosome and peptidases were significantly enriched (Supplementary Table 1).

### Relationship Between BMD and Microbial Community in Patients With PO

Since BMD is the basis for the diagnosis of osteoporosis and the severity of the disease can be judged, a correlation analysis was made between the microbial population change of the PO patient and BMD. For example, at the phylum level, Euryarchaeota and actinobacteria were negatively correlated with BMDh (*p* < 0.05), and at genus level, Haemophilus was negatively correlated with BMDs and BMDh (*p* < 0.05), Bifidobacterium was negatively correlated with BMDh (*p* < 0.05). However, based on the analysis of the key differential flora above, there was no significant difference in the flora associated with BMD at each taxonomic level between PO patients and HC groups (Supplementary Figure 1). Based on the correlation analysis between Alpha diversity and BMD, no significant correlation was found between BMD and intestinal microflora diversity index (*p* > 0.05).

### Predictive Performance of Gut Microbiome in Patients With PO

**Figure 5:**
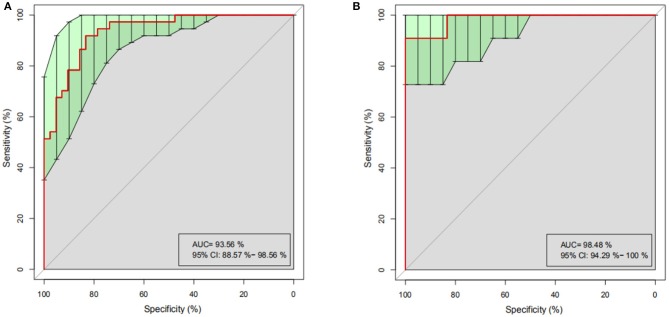
Random Forest analysis. Based on the representative discriminant model of 20 dominant genera, PO and HC are effectively distinguished. **(A)** ROC Curve of training set, abscissa: specificity scale, ordinate: sensitivity scale. **(B)** ROC curve of test set, abscissa: specificity scale, ordinate: sensitivity scale. ROC, receiver operating characteristic; AUC, area under the ROC curve; CI, confidence interval.

To determine whether differences in gut microbiome composition can be regarded as recognition biomarkers for distinguishing HC from PO patients, important species were selected by MeanDecreaseAccuracy and MeanDecreaseGin (Supplementary Figure 2). Based on the level of species abundance, different number of species were selected according to gradient to conduct random forest analyses. Random forest model was generated and ROC curve was drawn to evaluate its distinguishing ability. As shown in Figure 5A, the discriminant model based on the representative 20 dominant genera effectively distinguishes PO from HC (AUC = 93.56%, 95% CI: 88.57–98.56%), after cross-validation of the model, the analysis of intestinal microbiota in the verification groups showed a relatively high accuracy of diagnosis (AUC = 98.48, 95% CI: 94.29–100%) (Figure 5B). These results show that the random forest model based on fecal microflora can distinguish PO patients from healthy individuals among Chinese population, indicating that intestinal microflora information can be used to identify PO patients.

## Discussion

In this study, we found that the abundance of gut microbiome in patients with PO was uncommonly higher than that in subjects with HC, which was consistent with the results of many previous studies, and supported the view that excessive growth of intestinal bacteria would lead to bone loss (Di Stefano et al., 2001; Stotzer et al., 2003). PO is a complex disease affected by multiple risk factors, including genetic factors and environmental factors (Ensrud and Crandall, 2017). More and more studies have shown that the intestinal flora may regulate the immune system and endocrine system through the gut–brain axis to participate in the body's bone metabolism. The imbalance of gut microbiome can cause the imbalance of osteogenesis and osteoclast reaction and cause osteopenia, while the specific mechanism has not been determined (Ohlsson and Sjögren, 2015; Yan and Charles, 2017; Ibáñez et al., 2019). As far as we know, there are few studies on the relationship between intestinal flora and osteoporosis, we characterized the disease-related microbiota by 16s rDNA Amplicon Sequencing, proving that there are possible biomarkers (including microbial composition and function) in gut microbiome group in patients with PO. Notably, the discriminant model based on dominant microflora can effectively distinguish PO patients from healthy individuals. The new results of this study will provide new clues to understand the disease, provide markers for the diagnosis of the disease, and give new strategies for intervention treatment.

Alpha Diversity revealed that significant changes in the structure of gut microbiome were found in patients with PO at a wide range of phylogenetic levels. The result show that the bacterial community was mainly distributed in Firmicutes, Bacteroidetes, Proteobacteria, and Actinomyces. Interestingly, Bacteroidetes was significantly enriched in PO group. At the genus level, Faecalibacterium, Sundoligranulum, Agathobacter are relatively abundant bacterial communitys in both PO group and HC group. Previous studies had found that there is a negative correlation between Ratio of Firmicutes/Bacteriodetes and bone mass (Eckburg et al., 2005; Qin et al., 2010), which is consistant with our results support. Wang found that the abundance of Firmicutes flora in patients with osteoporosis decreased significantly, the result is similar to ours and support the findings of Eckburg and Qin. Moreover, Bacteroides, Faecalibacterium, and Prevotella contributed more than half of the bacterial community in the HC group at genus level (Wang et al., 2017). Compared with our results, there are some differences in the relatively abundant flora in the HC group. By comparing the gut microbiome gene pool between PO patients and HC group, we found that Bacteroidetes, Ruminococcaceae family, Prevotellaceae family, Faecalibacterium genus, and Dialister genus played a key role in the change of intestinal flora in PO patients. What is of concern is that the abundance of Faecalibacterium and Dialister is significantly increased in patients with PO. More importantly, the model based on these two dominant bacteria can distinguish PO patients from HC. Faecalibacterium is one of the most abundant bacteria in the intestinal tract, one of the main producers of butyric acid in the intestinal tract, which play an important role in promoting intestinal health has been (Belzer et al., 2017). Interestingly, butyric acid is a type of short-chain fatty acid that can increase calcium deposition in osteoblasts by affecting the Runx and osteoprotegerin signaling pathways and stimulate bone formation by increasing the production of bone sialoprotein and osteopontin (Katono et al., 2008; Tyagi et al., 2018), can also stimulate the expression of osteoblast gene, promote the differentiation of osteoblasts and inhibit the differentiation of osteoclasts (Lee et al., 2006; Lucas et al., 2018). Butyric acid may also indirectly affect BMD by affecting the function of endocrine factors related to bone metabolism (Kanda et al., 2017; Tang et al., 2017). Therefore, Faecalibacterium can regulate bone metabolism by producing butyric acid, promote bone formation, and inhibit bone resorption. In addition, studies have shown that inflammatory cytokines produced by activated T cells can increase osteoclast activity and lead to bone resorption in autoimmune diseases such as rheumatoid arthritis (Biver et al., 2019). Faecalibacterium has anti-inflammatory properties, which can induce the increase of anti-inflammatory cytokine IL-10 and other cytokines, thus inhibiting bone resorption (Miquel et al., 2013). Obviously, the decrease of bone mass in patients with PO is not caused by the abundance of Faecalibacterium (Wang et al., 2016). Gut microbiome interact with the human body from the initial stage of life, which is affected by many factors, such as host genetics, diet, age, geography, host immune status, travel, and the use of certain drugs. Therefore, we surmise that the increase of Faecalibacterium abundance in patients with PO may be affected by the decrease of host bone mineral density and the feedback regulation made by the body in order to balance bone metabolism (Jandhyala, 2015). Dialister is an anaerobic Gram-negative bacilli, which is associated with a variety of human infectious diseases, mainly related to oral infectious diseases. It is worth noting that some studies have found that Dialister may be related to the decrease of alveolar bone mass (Slots et al., 2002; Jumas-Bilak, 2005; Morio et al., 2007). In addition, the abundance of Dialister flora was significantly enriched with the increase of interleukin (IL)-6 level (Martínez et al., 2013). IL-6 plays a key role in bone metabolism, and the increase of IL-6 level leads to bone loss. This may explain the increase of Dialister flora abundance in patients with PO (Rossi et al., 2019). In addition, the abundance of Dialister decreased in patients with rheumatoid arthritis and upper motor neuron intestinal syndrome after spinal cord injury (Gungor et al., 2016; Tito et al., 2017). Of course, the above assumptions are our conjectures and need to be further studied and demonstrated. Moreover, the interaction between Faecalibacterium and Dialister in human body needs to be fully studied.

In addition, with the continuous study of gut-brain axis, it has been found that gut microbiome may interact with the bone metabolism of the host through a variety of potential pathways (Yan and Charles, 2017). Meta-genome function prediction results show that PO is related to the extensive regulation of functional KEGG pathway, mainly involved in genetic information processing, metabolism, environmental information processing, cellular processes, human diseases, and organic systems. Previous studies have shown that gut microbiome has a close effect on host bone metabolism through metabolism, endocrine, and immune communication (Hernandez et al., 2016). Our results support this conclusion: we found that the endocrine system, immune system, digestive system and immune system disease microbial function in PO patients with grade 2 KEGG signaling pathway were significantly higher than those in HC group. what is important is that, Membrane Transport modules and Carbohydrate Metabolism modules are vital components, and these functional modules are significantly reduced in PO patients. Membrane Transport module is closely related to cell life, and cell life depends to a large extent on the ability of the membrane to accurately control the solute exchange between the internal and external (environmental) regions. The decrease of membrane transport functional modules may lead to premature apoptosis (Kulbacka et al., 2017). At the same time, membrane transport involves the complex mechanism of passive and active transport of ions and small and medium-sized molecules, and its disorder may affect the metabolism of calcium salts, thus affecting bone metabolism (Pavlos and Friedman, 2017). Carbohydrate metabolism module is the basis of host metabolism mechanism. Carbohydrate metabolism is carried out through glycolysis pathway, pentose phosphate pathway, and anaerobic carbohydrate decomposition pathway, through which, complex carbohydrates are absorbed into simple carbohydrates. These sugars are further fermented into short-chain fatty acids, including butyric acid, which provide an important source of nutrition and regulatory control of the host digestive system (LeBlanc et al., 2017). Butyric acid has an important effect on bone metabolism and can promote bone formation and inhibit bone resorption (Katono et al., 2008). In addition, carbohydrate metabolism module can also affect the production of probiotic globulin on related hormones such as glucagon-like peptide-1 and peptide-yy, so we can infer that the decrease of carbohydrate metabolism module has a close causal relationship with PO (Clarke et al., 2014). Vitamin D promotes calcium absorption and plays a key role in bone metabolism. Previous studies have shown that gut microflora can affect vitamin D metabolism (Szaleniec et al., 2018). We found that there was no significant difference in the strains which affect the metabolism of vitamin D between the two groups. Therefore, the relationship between vitamin D and intestinal microbiota needs to be further explored.

In this study, we proved that the abundance of gut microbiome community in patients with PO was significantly enriched, and the key flora leading to the difference was found. It is worth noting that the model based on these dominant flora can distinguish PO patients from healthy individuals among Chinese population. We also deduced the possible mechanism of PO from the prediction of metagenomic function of gut microbiome in patients with PO, which is very important in the field of PO research. Because the pathophysiological process of PO patients began many years before diagnosis, it is of great significance to prevent the occurrence of PO. At the same time, it also provides a new target for the treatment of PO.

We tried to design a perfect research, but there are still some defects. Firstly, although the participants of two groups had the same eating habits, there also were great differences in specific recipes, which may interfere with the composition and structure of gut microbiome. Secondly, further longitudinal studies are needed to determine the causal relationship between gut microbiome and PO. Lastly, our research methods also have some limitations. 16s rDNA sequencing analysis has the characteristics of insufficient depth of species identification and can not be distinguished to the level of species. In addition, the PICRUSt prediction process will cause part of the loss of the original data, and the prediction results of flora from different sources are also different. It can only predict the known functions of known microorganisms. The above shortcomings need to be further improved by macro-genome sequencing analysis. Despite these limitations, our research provides an important introduction to the gut microbiome of PO patients and may have important clinical significance for the prevention and treatment of PO.

## Data Availability Statement

The datasets generated for this study can be found in the National Center for Biotechnology Information (NCBI) Bioprjoect database with project number PRJNA590676, https://www.ncbi.nlm.nih.gov/sra/?term=PRJNA590676.

## Ethics Statement

The studies involving human participants were reviewed and approved by Ethics Committee of the First Affiliated Hospital of Zhengzhou University. The patients/participants provided their written informed consent to participate in this study.

## Author Contributions

GP and FL designed the study. ZXu and ZXi analyzed the data, created figures, and wrote the manuscript. JS and SH collected clinical data. YC, CL, XS, BX, LT, and CG collected stool samples. All authors read and approved the final manuscript.

## Conflict of Interest

The authors declare that the research was conducted in the absence of any commercial or financial relationships that could be construed as a potential conflict of interest.
