# Supplementary material for: Gut Microbiome Reveals Specific Dysbiosis in Primary Osteoporosis
Source: Front Cell Infect Microbiol. 2020 Apr 21;10:160. doi: 10.3389/fcimb.2020.00160 (PMC7186314; doi:10.3389/fcimb.2020.00160)
Supplement: Supplementary file 1 [file Data_Sheet_1.docx]

Supplementary Material

# Supplementary Tables

| Supplementary Table S1. Functional pathways differences at level 2 inferred from 16S rRNA gene sequences using PICRUSt. | | | | | | | | | |
| --- | --- | --- | --- | --- | --- | --- | --- | --- | --- |
| KO functional categories | | |  | PO |  |  | HC | |  |
| Level_1 | Level_2 | Level_3 |  | mean% | SD% |  | mean% | SD% | P-value |
| Environmental Information Processing | Membrane Transport | Transporters |  | 0.072645 | 0.007932 |  | 0.08038 | 0.00937 | <0.001 |
| Environmental Information Processing | Membrane Transport | ABC transporters |  | 0.036335 | 0.004046 |  | 0.038695 | 0.004521 | 0.008 |
| Unclassified | Poorly Characterized | General function prediction only |  | 0.035646 | 0.001376 |  | 0.03502 | 0.001232 | 0.021 |
| Genetic Information Processing | Replication and Repair | DNA repair and recombination roteins |  | 0.027504 | 0.001527 |  | 0.026628 | 0.001922 | 0.015 |
| Genetic Information Processing | Translation | Ribosome |  | 0.023159 | 0.002092 |  | 0.021678 | 0.002537 | 0.002 |
| Genetic Information Processing | Transcription | Transcription factors |  | 0.018883 | 0.001902 |  | 0.020149 | 0.002001 | 0.002024 |
| Metabolism | Enzyme Families | Peptidases |  | 0.018743 | 0.000917 |  | 0.018248 | 0.000856 | 0.007 |
| Metabolism | Nucleotide Metabolism | Pyrimidine metabolism |  | 0.018152 | 0.001257 |  | 0.017304 | 0.001472 | 0.003 |
| Genetic Information Processing | Replication and Repair | Chromosome |  | 0.016372 | 0.000883 |  | 0.015532 | 0.001366 | <0.001 |
| Metabolism | Amino Acid Metabolism | Amino acid related enzymes |  | 0.015033 | 0.000848 |  | 0.014364 | 0.001109 | 0.001 |
| Genetic Information Processing | Translation | Ribosome Biogenesis |  | 0.014793 | 0.000746 |  | 0.014243 | 0.001105 | 0.005 |
| Metabolism | Amino Acid Metabolism | Arginine and proline metabolism |  | 0.012723 | 0.000593 |  | 0.012418 | 0.000787 | 0.034 |
| Metabolism | Energy Metabolism | Methane metabolism |  | 0.011665 | 0.000996 |  | 0.012452 | 0.001156 | <0.001 |
| Genetic Information Processing | Replication and Repair | DNA replication proteins |  | 0.012244 | 0.000854 |  | 0.011672 | 0.001116 | 0.005 |
| Metabolism | Amino Acid Metabolism | Alanine, aspartate and glutamate metabolism |  | 0.010957 | 0.000787 |  | 0.010399 | 0.000938 | 0.002 |
| Metabolism | Amino Acid Metabolism | Cysteine and methionine metabolism |  | 0.00991 | 0.00035 |  | 0.009637 | 0.00042 | <0.001 |
| Genetic Information Processing;; | Folding, Sorting and Degradation | Chaperones and folding catalysts |  | 0.009884 | 0.000674 |  | 0.009201 | 0.0007 | <0.001 |
| Metabolism | Carbohydrate Metabolism | Fructose and mannose metabolism |  | 0.008866 | 0.001022 |  | 0.010167 | 0.002867 | 0.004 |
| Genetic Information Processing | Transcription | Transcription machinery |  | 0.010062 | 0.000992 |  | 0.009431 | 0.001339 | 0.010 |
| Metabolism | Carbohydrate Metabolism | Pentose phosphate pathway |  | 0.00921 | 0.000502 |  | 0.009757 | 0.001128 | 0.003 |
| Unclassified | Metabolism | Others |  | 0.008659 | 0.000757 |  | 0.009562 | 0.000677 | <0.001 |
| Unclassified | Genetic Information Processing | Translation proteins |  | 0.009212 | 0.000461 |  | 0.008807 | 0.000608 | <0.001 |
| Genetic Information Processing | Replication and Repair | Homologous recombination |  | 0.008986 | 0.000588 |  | 0.00865 | 0.000815 | 0.022 |
| Unclassified | Metabolism | Energy metabolism |  | 0.008702 | 0.000717 |  | 0.007865 | 0.000646 | <0.001 |
| Metabolism | Energy Metabolism | Carbon fixation pathways in prokaryotes |  | 0.008544 | 0.000892 |  | 0.00789 | 0.000753 | <0.001 |
| Metabolism | Glycan Biosynthesis and Metabolism | Peptidoglycan biosynthesis |  | 0.008364 | 0.000602 |  | 0.008058 | 0.000744 | 0.029 |
| Metabolism | Carbohydrate Metabolism | Butanoate metabolism |  | 0.006068 | 0.000693 |  | 0.005764 | 0.00077 | 0.045 |
| Environmental Information Processing | Membrane Transport | Bacterial secretion system |  | 0.005955 | 0.000804 |  | 0.005546 | 0.001044 | 0.034 |
| Metabolism | Metabolism of Cofactors and Vitamins | One carbon pool by folate |  | 0.006151 | 0.000516 |  | 0.00569 | 0.000527 | <0.001 |
| Genetic Information Processing | Folding, Sorting and Degradation | Protein export |  | 0.005841 | 0.000419 |  | 0.005546 | 0.000441 | 0.001 |
| Metabolism | Lipid Metabolism | Lipid biosynthesis proteins |  | 0.005779 | 0.000304 |  | 0.005292 | 0.000487 | <0.001 |
| Environmental Information Processing | Membrane Transport | Phosphotransferase system (PTS) |  | 0.003905 | 0.001713 |  | 0.006098 | 0.004539 | 0.002 |
| Metabolism | Metabolism of Terpenoids and Polyketides | Terpenoid backbone biosynthesis |  | 0.005732 | 0.00048 |  | 0.005376 | 0.000591 | 0.002 |
| Metabolism | Metabolism of Cofactors and Vitamins | Thiamine metabolism |  | 0.005546 | 0.000456 |  | 0.005264 | 0.000721 | 0.024 |
| Genetic Information Processing | Translation | Translation factors |  | 0.005237 | 0.000392 |  | 0.004896 | 0.00047 | <0.001 |
| Metabolism | Energy Metabolism | Photosynthesis proteins |  | 0.005079 | 0.000638 |  | 0.004607 | 0.000937 | 0.005 |
| Metabolism | Energy Metabolism | Photosynthesis |  | 0.005054 | 0.000661 |  | 0.00458 | 0.000965 | 0.006 |
| Cellular Processes | Cell Growth and Death | Cell cycle - Caulobacter |  | 0.004925 | 0.000577 |  | 0.004696 | 0.000527 | 0.045 |
| Metabolism | Carbohydrate Metabolism | Citrate cycle (TCA cycle) |  | 0.004875 | 0.000864 |  | 0.004418 | 0.000767 | 0.007 |
| Genetic Information Processing | Folding, Sorting and Degradation | RNA degradation |  | 0.004589 | 0.00027 |  | 0.00426 | 0.000336 | <0.001 |
| Metabolism | Metabolism of Cofactors and Vitamins | Nicotinate and nicotinamide metabolism |  | 0.004256 | 0.000213 |  | 0.004144 | 0.000295 | 0.036 |
| Metabolism | Lipid Metabolism | Glycerolipid metabolism |  | 0.003848 | 0.00042 |  | 0.004361 | 0.000415 | <0.001 |
| Genetic Information Processing | Replication and Repair | Nucleotide excision repair |  | 0.003886 | 0.000362 |  | 0.003692 | 0.000455 | 0.023 |
| Metabolism | Enzyme Families | Protein kinases |  | 0.003151 | 0.000415 |  | 0.003486 | 0.000548 | 0.001 |
| Metabolism | Carbohydrate Metabolism | C5-Branched dibasic acid metabolism |  | 0.003243 | 0.000285 |  | 0.003409 | 0.000364 | 0.014 |
| Metabolism | Metabolism of Terpenoids and Polyketides | Prenyltransferases |  | 0.002855 | 0.000286 |  | 0.002654 | 0.000241 | <0.001 |
| Metabolism | Metabolism of Cofactors and Vitamins | Riboflavin metabolism |  | 0.002451 | 0.00026 |  | 0.002127 | 0.00035 | <0.001 |
| Metabolism | Biosynthesis of Other Secondary Metabolites | Phenylpropanoid biosynthesis |  | 0.002047 | 0.000581 |  | 0.002294 | 0.000555 | 0.036 |
| Unclassified | Metabolism | Carbohydrate metabolism |  | 0.001746 | 0.000358 |  | 0.001926 | 0.00029 | 0.007 |
| Metabolism | Metabolism of Cofactors and Vitamins | Vitamin B6 metabolism |  | 0.001966 | 0.000148 |  | 0.001832 | 0.000178 | <0.001 |
| Unclassified | Genetic Information Processing | Restriction enzyme |  | 0.002075 | 0.000347 |  | 0.001765 | 0.000433 | <0.001 |
| Unclassified | Metabolism | Amino acid metabolism |  | 0.00152 | 0.000339 |  | 0.00178 | 0.000573 | 0.009 |
| Metabolism | Xenobiotics Biodegradation and Metabolism | Chloroalkane and chloroalkene degradation |  | 0.001481 | 0.000745 |  | 0.001735 | 0.000449 | 0.047 |
| Human Diseases | Infectious Diseases | Tuberculosis |  | 0.001649 | 0.00016 |  | 0.001501 | 0.000239 | 0.000612 |
| Genetic Information Processing | Translation | RNA transport |  | 0.001379 | 0.000246 |  | 0.001553 | 0.000229 | 0.000562 |
| Metabolism | Metabolism of Terpenoids and Polyketides | Biosynthesis of ansamycins |  | 0.001374 | 0.000199 |  | 0.001503 | 0.000261 | 0.008 |
| Cellular Processes | Transport and Catabolism | Peroxisome |  | 0.001554 | 0.000224 |  | 0.00132 | 0.000202 | <0.001 |
| Metabolism | Metabolism of Other Amino Acids | D-Glutamine and D-glutamate metabolism |  | 0.001453 | 9.28E-05 |  | 0.001346 | 0.000118 | <0.001 |
| Metabolism | Biosynthesis of Other Secondary Metabolites | Novobiocin biosynthesis |  | 0.001402 | 0.000192 |  | 0.001273 | 0.000127 | <0.001 |
| Metabolism | Metabolism of Terpenoids and Polyketides | Tetracycline biosynthesis |  | 0.001113 | 0.000291 |  | 0.001241 | 0.000336 | 0.048 |
| Metabolism | Biosynthesis of Other Secondary Metabolites | Tropane, piperidine and pyridine alkaloid biosynthesis |  | 0.001133 | 0.000218 |  | 0.001033 | 0.000122 | 0.006 |
| Metabolism | Carbohydrate Metabolism | Ascorbate and aldarate metabolism |  | 0.000725 | 0.000474 |  | 0.001088 | 0.000758 | 0.006 |
| Metabolism | Xenobiotics Biodegradation and Metabolism | Naphthalene degradation |  | 0.000937 | 0.000269 |  | 0.001066 | 0.000301 | 0.029 |
| Environmental Information Processing | Signaling Molecules and Interaction | Bacterial toxins |  | 0.000906 | 0.000201 |  | 0.001045 | 0.000246 | 0.003 |
| Metabolism | Metabolism of Other Amino Acids | Taurine and hypotaurine metabolism |  | 0.00096 | 9.71E-05 |  | 0.000903 | 0.000122 | 0.013 |
| Organismal Systems | Endocrine System | PPAR signaling pathway |  | 0.001071 | 0.000237 |  | 0.000908 | 0.000175 | <0.001 |
| Metabolism | Carbohydrate Metabolism | Inositol phosphate metabolism |  | 0.000747 | 0.000189 |  | 0.000887 | 0.000336 | 0.014 |
| Metabolism | Xenobiotics Biodegradation and Metabolism | Toluene degradation |  | 0.000753 | 0.000436 |  | 0.000559 | 0.000365 | 0.020 |
| Metabolism | Metabolism of Terpenoids and Polyketides | Limonene and pinene degradation |  | 0.000504 | 0.000237 |  | 0.000621 | 0.000305 | 0.038 |
| Metabolism | Xenobiotics Biodegradation and Metabolism | Dioxin degradation |  | 0.000436 | 0.000155 |  | 0.000611 | 0.00021 | <0.001 |
| Metabolism | Xenobiotics Biodegradation and Metabolism | Xylene degradation |  | 0.000417 | 0.000128 |  | 0.000577 | 0.00017 | <0.001 |
| Organismal Systems | Endocrine System | Adipocytokine signaling pathway |  | 0.00063 | 0.000214 |  | 0.000465 | 0.000176 | <0.001 |
| Metabolism | Biosynthesis of Other Secondary Metabolites | Isoquinoline alkaloid biosynthesis |  | 0.00051 | 9.82E-05 |  | 0.000429 | 9.78E-05 | <0.001 |
| Genetic Information Processing | Translation | Ribosome biogenesis in eukaryotes |  | 0.000481 | 4.47E-05 |  | 0.000459 | 5.40E-05 | 0.036 |
| Human Diseases | Metabolic Diseases | Type I diabetes mellitus |  | 0.000487 | 4.94E-05 |  | 0.000454 | 5.96E-05 | 0.005 |
| Metabolism | Metabolism of Terpenoids and Polyketides | Zeatin biosynthesis |  | 0.000495 | 7.37E-05 |  | 0.000441 | 5.90E-05 | <0.001 |
| Human Diseases | Immune System Diseases | Primary immunodeficiency |  | 0.000389 | 0.000139 |  | 0.00045 | 0.000124 | 0.025 |
| Human Diseases | Cancers | Pathways in cancer |  | 0.00046 | 6.48E-05 |  | 0.000438 | 4.18E-05 | 0.045 |
| Organismal Systems | Immune System | NOD-like receptor signaling pathway |  | 0.000443 | 7.70E-05 |  | 0.000411 | 6.32E-05 | 0.026 |
| Metabolism | Lipid Metabolism | Primary bile acid biosynthesis |  | 0.000373 | 0.000128 |  | 0.000435 | 0.00013 | 0.020 |
| Metabolism | Lipid Metabolism | Secondary bile acid biosynthesis |  | 0.000371 | 0.000128 |  | 0.000432 | 0.00013 | 0.022 |
| Metabolism | Xenobiotics Biodegradation and Metabolism | Styrene degradation |  | 0.000111 | 7.20E-05 |  | 0.000179 | 0.000157 | 0.008 |
| Metabolism | Xenobiotics Biodegradation and Metabolism | Atrazine degradation |  | 0.000118 | 8.07E-05 |  | 0.000165 | 0.000139 | 0.046 |
| Environmental Information Processing | Signaling Molecules and Interaction | Cellular antigens |  | 0.000203 | 0.000222 |  | 0.000107 | 0.000112 | 0.009 |
| Metabolism | Biosynthesis of Other Secondary Metabolites | Flavone and flavonol biosynthesis |  | 0.000168 | 6.29E-05 |  | 0.000107 | 6.21E-05 | <0.001 |
| Metabolism | Lipid Metabolism | Arachidonic acid metabolism |  | 0.000148 | 0.000151 |  | 8.53E-05 | 0.00011 | 0.023 |
| Genetic Information Processing | Replication and Repair | Non-homologous end-joining |  | 3.73E-05 | 2.73E-05 |  | 8.19E-05 | 0.000141 | 0.036 |
| Organismal Systems | Digestive System | Protein digestion and absorption |  | 9.72E-05 | 0.000148 |  | 3.88E-05 | 5.58E-05 | 0.013 |
| Human Diseases | Infectious Diseases | Amoebiasis |  | 3.77E-05 | 2.37E-05 |  | 5.82E-05 | 5.68E-05 | 0.025 |
| Human Diseases | Neurodegenerative Diseases | Prion diseases |  | 2.82E-05 | 3.43E-05 |  | 4.77E-05 | 4.98E-05 | 0.028 |
| Genetic Information Processing | Transcription | Basal transcription factors |  | 5.43E-05 | 4.32E-05 |  | 1.95E-05 | 2.32E-05 | <0.001 |
| Metabolism | Lipid Metabolism | Ether lipid metabolism |  | 2.37E-05 | 2.09E-05 |  | 4.02E-05 | 5.02E-05 | 0.040 |
| Metabolism | Glycan Biosynthesis and Metabolism | Various types of N-glycan biosynthesis |  | 4.78E-07 | 9.60E-07 |  | 1.38E-07 | 3.10E-07 | 0.023 |
| Human Diseases | Infectious Diseases | Influenza A |  | 2.36E-07 | 6.81E-07 |  | 9.98E-09 | 2.22E-08 | 0.026 |
| Environmental Information Processing | Membrane Transport | Transporters |  | 0.072645 | 0.007932 |  | 0.08038 | 0.00937 | <0.001 |
| Environmental Information Processing | Membrane Transport | ABC transporters |  | 0.036335 | 0.004046 |  | 0.038695 | 0.004521 | 0.008 |
| Unclassified | Poorly Characterized | General function prediction only |  | 0.035646 | 0.001376 |  | 0.03502 | 0.001232 | 0.020 |

Note: P value is obtained by T-test. Data are given as mean%,SD%;Abbreviations: PO, primary osteoporosis;HC, normal cognition healthy control; KEGG, Kyoto Encyclopedia of Genes and Genomes; PICRUSt, Phylogenetic Investigation of Communities by Reconstruction of Unobserved States; KO, KEGG Ortholog; SD: standard deviation.

# Supplementary Figures


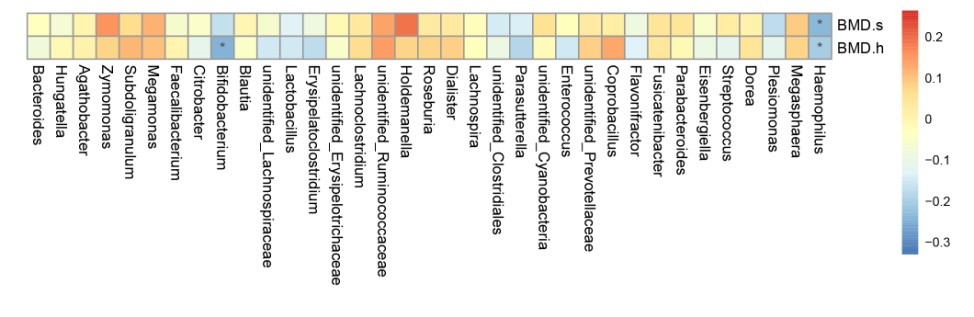


**Supplementary Figure S1.** Correlation analysis between bone mineral density and species richness at the genus level. Note: The longitudinal information is environmental factor information and the horizontal is species information. The corresponding value of intermediate heat map is Spearman correlation coefficient r, between-1, 1, r<0 is negative correlation, r>0 is positive correlation, marked * means significance test p<0. 05, and marked ** means significance test p<0. 01.


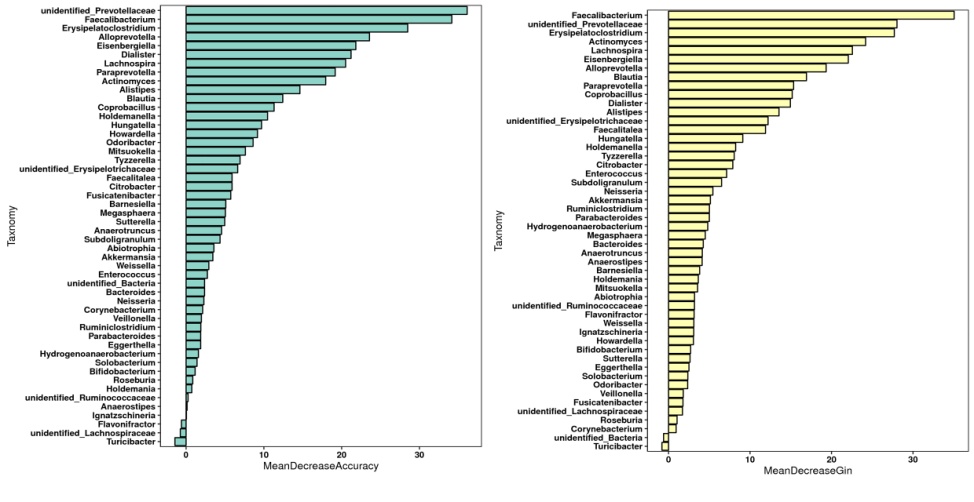


**Supplementary Figure S2.** Species importance ranking chart. MeanDecreaseAccuracy (left) measures the reduction in the accuracy of random forest prediction by changing the value of a variable into a random number. The higher the value, the more important the variable is. Abscissa: average decline accuracy, longitudinal coordinates: ranked at the top 50 important species; MeanDecreaseGini (right) calculates the influence of each variable on the heterogeneity of the observed values on each node of the classification tree through the Gini index, thus comparing the importance of the variables. The higher the value, the greater the importance of the variable is. Abscissa: average decline Gini index, ordinate: ranked at the top 50 important species.
